# Explore poverty with statistical modeling: The bivariate polynomial binary logit regression (BPBLR)

**DOI:** 10.1016/j.mex.2024.103099

**Published:** 2024-12-15

**Authors:** Vita Ratnasari, Purhadi, Marisa Rifada, Andrea Tri Rian Dani

**Affiliations:** Sepuluh Nopember Institute of Technology, Airlangga University, Mulawarman University, Indonesia

**Keywords:** Binary responses, Bivariate, Logit regression, Polynomial, Poverty, The Bivariate Polynomial Logit Regression Models

## Abstract

Logit regression (or logistic regression) is a statistical analysis of categorical data. The binary responses have two categories. We present the Bivariate Polynomial Binary Logit Regression (BPBLR), which extends logit regression by modeling two correlated binary response variables. This model uses a polynomial pattern to capture the association between the logit and predictor variables. The maximum likelihood estimation (MLE) method is used for parameter estimation, and the maximum likelihood ratio test (MLRT) method is used for the statistical testing of the proposed model. The distribution of the test statistics asymptotically is the Chi-square distribution. Selection of the optimal polynomial degree and the best model is based on the minimum Deviance value. Some highlights of the proposed method are:

• Statistical modeling innovation on categorical data with two correlated binary response variables, namely Bivariate Polynomial Binary Logit Regression (BPBLR).
• The statistical test is obtained using MLRT method.
• The BPBLR model is applied to actual datasets regarding the depth and severity of poverty to capture poverty problems SDGs 1

## Graphical abstract

Graphical abstract for the Bivariate Polynomial Binary Logit Regression (BPBLR).

**Figure:**
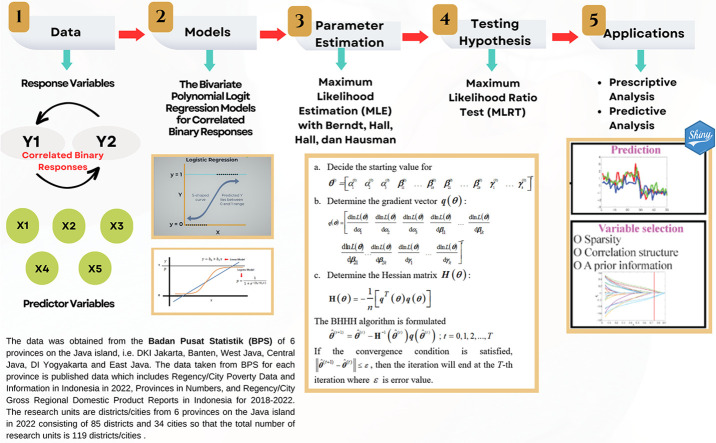

Specifications table

**Table:** 

Subject area:	Mathematics and Statistics
More specific subject area:	*Statistics; Regression; Economy, Poverty*
Name of your method:	*The Bivariate Polynomial Logit Regression Models*
Name and reference of original method:	1. M, Rifada, V. Ratnasari, and Purhadi, *Parameter Estimation and Hypothesis Testing of The Bivariate Polynomial Ordinal Logistic Regression Model*. Mathematics 2023, 11, 579. https://doi.org/10.3390/math11030579
	2. V. Ratnasari, S. H. Utama, and A. T. R. Dani, *Toward Sustainable Development Goals (SDGs) with Statistical Modeling: Recursive Bivariate Binary Probit*. IAENG International Journal of Applied Mathematics, vol. 54, no. 8, pp1515–1521, 2024.
	3. V. Ratnasari, Purhadi, I. C. Aviantholib, and A. T. R. Dani, “Parameter Estimation and Hypothesis Testing the Second Order of Bivariate Binary Logistic Regression (S-BBLR) Model with Berndt Hall-Hall-Hausman (BHHH) Iterations,” *Communications in Mathematical Biology and Neuroscience*, vol. 2022, 2022, doi: 10.28919/cmbn/7258.
Resource availability:	*There are two binary response variables and predictor variables in this study. The response variables are the depth of poverty (Y1) and the severity of poverty (Y2) in districts/cities on the Java-island in 2022. Each response variable has two categories (binary),* i.e. *low and high. The predictor variables are factors which is thought to influence the depth and severity of poverty, includes the open unemployment rate (X1), the literacy rate of the population aged ≥ 15 years (X2), the average number of years of schooling (X3), the middle age school participation (X4) and GDP growth rate at constant prices (X5). Recapitulation data from the Central Statistics Agency (BPS)*

## Background

One of the statistical techniques for categorical data is logit regression [[1], [2], [3], [4]]. Several studies on logit regression have been carried out for binary response variables, i.e., the binary logit regression [5,6]. Pregibon (1981) studied Maximum Likelihood Estimation (MLE) and numerical approaches such as Newton-Raphson and Iteratively Reweighted Least Square (IRLS) methods for parameter estimation of the binary logit model [7]. Parameter estimation, statistical testing, and selecting the optimal model were examined by Hosmer et al. [1]. In its development, several researchers extended binary logit regression to two correlated binary responses [8,9]. Palmgren (2011) introduced regression models for bivariate binary responses and examined parameter estimation using the MLE method [5]. Cessie and Houwelingen (1994) modeled logistic regression for correlated binary data, in which the data are modeled such that the marginal outcome probabilities are still logistic [9]. The previous study assumed that logit regression is “linear on the logit,” meaning that the logit and predictor variables are related linearly [10,11]. In fact, this assumption may be untenable in many areas of science [12].

According to Royston & Altman (1994), fractional polynomial can be used to analyze the functional form of a continuous predictor variable [13]. Several researchers have employed this approach, which features a more flexible association that can be either linear or non-linear [[14], [15], [16]]. Furthermore, several research studies have recently been conducted to investigate the usage of fractional polynomials for logit regression but are restricted to one binary response [[17], [18], [19]]. There are quite a few problems in logit regression modeling where the response variable involves two correlated response variables, so simultaneous analysis is required [20,21]. Therefore, this study extends logit regression with two correlated binary responses, and the association between the logit and predictor variables is represented in polynomials [22,23]. We refer to this model as the Bivariate Polynomial Binary Logit Regression (BPBLR) model. In this study, we used the MLE method for parameter estimation and the MLRT method for statistical testing of the BPBLR model [24]. Furthermore, the BPBLR model is applied to actual datasets.

The structure of this paper is as follows: The BPBLR model is stated in Method details. Method details explain the BPBLR model estimating parameter using the MLE and BHHH approaches and the statistical testing of the BPBLR model. An application of the BPBLR model is shown in Method Validation.

## Method details

### The bivariate polynomial binary logit regression (BPBLR) model

The bivariate binary logit regression is the logit regression with two correlated binary responses [8]. Suppose $Y_{1}$ and $Y_{2}$ are binary response variables consist of two categories with a value of 0 or 1, then the 2 × 2 contingency table for probability of the response is given in Table 1 as follows.

**Table 1:** The 2 × 2 contingency table.

	$Y_{2}=0$	$Y_{2}=1$	Total
$Y_{1}=0$	$\pi_{00}$	$\pi_{01}$	$1-\pi_{1}$
$Y_{1}=1$	$\pi_{10}$	$\pi_{11}$	$\pi_{1}$
Total	$1-\pi_{2}$	$\pi_{2}$	1

where $\pi_{gh}=P\left(Y_{1}=g,Y_{2}=h\right);g,h=0,1$ is the joint probability of the responses.

The BPBLR extends the bivariate binary logit regression by modeling a polynomial association between logit and predictor variables. Therefore, the BPBLR model can be expressed in Eq. (1).

(1) $$\begin{array}{ccc} \eta_{1} & = & g_{1}\left(x\right)=\text{logit}\left(\pi_{1}\left(x\right)\right)=ln\left(\frac{\pi_{1}\left(x\right)}{1-\pi_{1}\left(x\right)}\right)=\alpha_{1}+\sum_{j=1}^{k}\beta_{1j}^{T}x_{j\left(r_{j}\right)}^{*}\\ \eta_{2} & = & g_{2}\left(x\right)=\text{logit}\left(\pi_{2}\left(x\right)\right)=ln\left(\frac{\pi_{2}\left(x\right)}{1-\pi_{2}\left(x\right)}\right)=\alpha_{2}+\sum_{j=1}^{k}\beta_{2j}^{T}x_{j\left(r_{j}\right)}^{*}\\ \eta_{3} & = & g_{3}\left(x\right)=ln\left(\psi \left(x\right)\right)=ln\left(\frac{\pi_{00}\left(x\right)\pi_{11}\left(x\right)}{\pi_{01}\left(x\right)\pi_{10}\left(x\right)}\right)=\alpha_{3}+\sum_{j=1}^{k}\gamma_{j}^{T}x_{j\left(r_{j}\right)}^{*} \end{array}$$

where *k* represents the number of predictor variables, $\alpha_{1},\alpha_{2},\alpha_{3}$are the intercept parameter, whereas $\beta_{1j}$, $\beta_{2j}$and $\gamma_{j}$ are the parameters to be estimated and denoted by

$$\begin{array}{c} \beta_{1j}={\left[\begin{array}{cccc} \beta_{11j} & \beta_{21j} & \ldots & \beta_{r1j} \end{array}\right]}^{T}\\ \beta_{2j}={\left[\begin{array}{cccc} \beta_{12j} & \beta_{22j} & \ldots & \beta_{r2j} \end{array}\right]}^{T} \end{array}$$

$$\gamma_{2j}={\left[\begin{array}{cccc} \gamma_{1j} & \gamma_{2j} & \ldots & \gamma_{rj} \end{array}\right]}^{T}$$

x is the vector of covariates and denoted by $x={\left[\begin{array}{cccc} x_{1(r_{1})}^{*} & x_{2(r_{2})}^{*} & \ldots & x_{k(r_{k})}^{*} \end{array}\right]}^{T}$.

with $x_{j(r_{j})}^{*}={\left[\begin{array}{cccc} x_{j} & x_{j}^{2} & \ldots & x_{j}^{r_{j}} \end{array}\right]}^{T}$ is the vector of the *j*-th covariate on the *r*-th polynomial degree, $\pi_{1}\left(x\right)=P\left(Y_{1}=1|x\right)$ and $\pi_{2}\left(x\right)=P\left(Y_{2}=1|x\right)$ are marginal probability of $Y_{1}$ and $Y_{2}$, respectively. Based on Eq. (1), we get:

(2) $$\begin{array}{ccc} & & \pi_{1}\left(x\right)=\frac{exp\left(\alpha_{1}+\sum_{j=1}^{k}\beta_{1j}^{T}x_{j\left(r_{j}\right)}^{*}\right)}{1+exp\left(\alpha_{1}+\sum_{j=1}^{k}\beta_{1j}^{T}x_{j\left(r_{j}\right)}^{*}\right)}\\ & & \pi_{2}\left(x\right)=\frac{exp\left(\alpha_{2}+\sum_{j=1}^{k}\beta_{2j}^{T}x_{j\left(r_{j}\right)}^{*}\right)}{1+exp\left(\alpha_{2}+\sum_{j=1}^{k}\beta_{2j}^{T}x_{j\left(r_{j}\right)}^{*}\right)} \end{array}$$

According to [9], ψ(x) in Eq. (1) is the odds ratio, which indicates that the response variables are correlated. The formula of ψ(x) is defined as:

(3) $$\psi \left(x\right)=\frac{\pi_{00}\left(x\right)\pi_{11}\left(x\right)}{\pi_{01}\left(x\right)\pi_{10}\left(x\right)}$$

The joint probability, $\pi_{11}\left(x\right)$, can be obtained with the following formula:

(4) $$\pi_{11}\left(x\right)=\left\{ \begin{array}{cc} \frac{1}{2}{\left(\psi -1\right)}^{-1}\left(a-\sqrt{a^{2}+b}\right), & \psi \neq 1\\ \pi_{1}\left(x\right)\pi_{2}\left(x\right), & \psi =1 \end{array}\right.$$

with,

$a=1+\left(\psi -1\right)\left(\pi_{1}\left(x\right)+\pi_{2}\left(x\right)\right)$ and $b=-4\psi \left(\psi -1\right)\pi_{1}\left(x\right)\pi_{2}\left(x\right)$.

Furthermore, we can get

(5) $$\begin{array}{ccc} & & \pi_{10}\left(x\right)=\pi_{1}\left(x\right)-\pi_{11}\left(x\right)\\ & & \pi_{01}\left(x\right)=\pi_{2}\left(x\right)-\pi_{11}\left(x\right)\\ & & \pi_{00}\left(x\right)=1-\pi_{1}\left(x\right)-\pi_{2}\left(x\right)+\pi_{11}\left(x\right) \end{array}$$

### Estimating the BPBLR model parameter

The estimated parameter in the BPBLR model is obtained by the MLE method. The first step is by taking *n* independent random samples so that the random variables $Y_{11i},Y_{10i},Y_{01i},Y_{00i}$ having a Multinomial distribution with each probability is $\pi_{11i},\pi_{10i},\pi_{01i},\pi_{00i}$. The Multinomial distribution probability of random variable $\left(Y_{1i},Y_{2i}\right)$ is:

(6) $$\begin{array}{ccc} & & P\left(Y_{11i}=y_{11i},Y_{10i}=y_{10i},Y_{01i}=y_{01i},Y_{00i}=y_{00i}\right)\\ & & \;\;=\pi_{11i}^{y_{11i}}\pi_{10i}^{y_{10i}}\pi_{01i}^{y_{01i}}\pi_{00i}^{y_{00i}} \end{array}$$

Based on Eq. (6), the likelihood function for the BPBLR model is:

(7) $$L\left(\theta \right)=\prod_{i=1}^{n}\left(\pi_{11i}^{y_{11i}}\pi_{10i}^{y_{10i}}\pi_{01i}^{y_{01i}}\pi_{00i}^{y_{00i}}\right)$$

with:

$\theta ={\left[\begin{array}{c} \alpha_{1}\;\alpha_{2}\;\alpha_{3}\;\beta_{11}\;\ldots \;\beta_{1k}\;\beta_{21}\;\ldots \;\beta_{2k}\;\gamma_{1}\;\ldots \;\gamma_{k} \end{array}\;\;\;\right]}^{T}$ is the parameter of the BPBLR model. Furthermore, we have the natural logarithm likelihood function in Eq. (8).

(8) $$\ln L\left(\theta \right)=\sum_{i=1}^{n}\left(y_{11i}\ln\pi_{11i}+y_{10i}\ln\pi_{10i}+y_{01i}\ln\pi_{01i}+y_{00i}\ln\pi_{00i}\right)$$

Substitute Eq. (5) to Eq. (8), then we have the following ln-likelihood function in Eq. (9)

(9) $$\ln L\left(\theta \right)=\sum_{i=1}^{n}\left[y_{11i}\ln\pi_{11i}+y_{10i}\ln\left(\pi_{1i}-\pi_{11i}\right)+y_{01i}\ln\left(\pi_{2i}-\pi_{11i}\right)+y_{00i}\ln\left(1-\pi_{1i}-\pi_{2i}+\pi_{11i}\right)\right]$$

The next step is maximizing the ln-likelihood function. The following is the first partial derivative of Eq. (10):

(10) $$\begin{array}{ccc} & & \frac{d\ln L\left(\theta \right)}{d\alpha_{1}}=\sum_{i=1}^{n}\frac{1}{\Delta_{1i}}\left(\frac{y_{11i}\pi_{01i}-y_{01i}\pi_{11i}}{\pi_{2i}}+\frac{y_{10i}\pi_{00i}-y_{00i}\pi_{10i}}{1-\pi_{2i}}\right)\\ & & \frac{d\ln L\left(\theta \right)}{d\beta_{1j}}=\sum_{i=1}^{n}\frac{1}{\Delta_{1i}}\left(\frac{y_{11i}\pi_{01i}-y_{01i}\pi_{11i}}{\pi_{2i}}+\frac{y_{10i}\pi_{00i}-y_{00i}\pi_{10i}}{1-\pi_{2i}}\right)x_{j\left(r_{j}\right)}^{*}\\ & & \frac{d\ln L\left(\theta \right)}{d\alpha_{2}}=\sum_{i=1}^{n}\frac{1}{\Delta_{1i}}\left(\frac{y_{11i}\pi_{10i}-y_{10i}\pi_{11i}}{\pi_{1i}}+\frac{y_{01i}\pi_{00i}-y_{00i}\pi_{01i}}{1-\pi_{1i}}\right)\\ & & \frac{d\ln L\left(\theta \right)}{d\beta_{2j}}=\sum_{i=1}^{n}\frac{1}{\Delta_{1i}}\left(\frac{y_{11i}\pi_{10i}-y_{10i}\pi_{11i}}{\pi_{1i}}+\frac{y_{01i}\pi_{00i}-y_{00i}\pi_{01i}}{1-\pi_{1i}}\right)x_{j\left(r_{j}\right)}^{*}\\ & & \frac{d\ln L\left(\theta \right)}{d\alpha_{3}}=\sum_{i=1}^{n}\Delta_{2i}\left(\frac{y_{11i}}{\pi_{11i}}-\frac{y_{10i}}{\pi_{10i}}-\frac{y_{01i}}{\pi_{01i}}+\frac{y_{00i}}{\pi_{00i}}\right)\\ & & \frac{d\ln L\left(\theta \right)}{d\gamma_{j}}=\sum_{i=1}^{n}\Delta_{2i}\left(\frac{y_{11i}}{\pi_{11i}}-\frac{y_{10i}}{\pi_{10i}}-\frac{y_{01i}}{\pi_{01i}}+\frac{y_{00i}}{\pi_{00i}}\right)x_{j\left(r_{j}\right)}^{*} \end{array}$$

with,

(11) $$\begin{array}{ccc} & & \Delta_{1i}=\frac{\pi_{11i}\pi_{10i}\pi_{01i}\pi_{00i}}{\pi_{1i}\left(1-\pi_{1i}\right)\pi_{2i}\left(1-\pi_{2i}\right){\left(\frac{1}{\pi_{11i}}+\frac{1}{\pi_{10i}}+\frac{1}{\pi_{01i}}+\frac{1}{\pi_{00i}}\right)}^{-1}}\\ & & \Delta_{2i}={\left(\frac{1}{\pi_{11}}+\frac{1}{\pi_{10}}+\frac{1}{\pi_{01}}+\frac{1}{\pi_{00}}\right)}^{-1} \end{array}$$

The result of Eq. (10) are non explicit form; so, the maximum likelihood estimators is obtained by numerical iteration. We use the BHHH [20] iteration as follows:

a. Decide the starting value for
  $$\theta^{\left(0\right)}={\left[\begin{array}{cccccccccccc} \alpha_{1}^{\left(0\right)} & \alpha_{2}^{\left(0\right)} & \alpha_{3}^{\left(0\right)} & \beta_{11}^{\left(0\right)} & \ldots & \beta_{1k}^{\left(0\right)} & \beta_{21}^{\left(0\right)} & \ldots & \beta_{2k}^{\left(0\right)} & \gamma_{1}^{\left(0\right)} & \ldots & \gamma_{k}^{\left(0\right)} \end{array}\right]}^{T}$$
b. Determine the gradient vector q(θ)
  $$\begin{array}{c} q\left(\theta \right)=[\;\;\;\begin{array}{cccccccc} \frac{d\ln L\left(\theta \right)}{d\alpha_{1}} & \frac{d\ln L\left(\theta \right)}{d\alpha_{2}} & \frac{d\ln L\left(\theta \right)}{d\alpha_{3}} & \frac{d\ln L\left(\theta \right)}{d\beta_{11}} & \ldots & \frac{d\ln L\left(\theta \right)}{d\beta_{1k}} & & \end{array}\\ \frac{d\ln L\left(\theta \right)}{d\beta_{21}}\ldots {\begin{array}{cccc} \frac{d\ln L\left(\theta \right)}{d\beta_{2k}} & \frac{d\ln L\left(\theta \right)}{d\gamma_{1}} & \ldots & \frac{d\ln L\left(\theta \right)}{d\gamma_{k}} \end{array}\;\;\;]}^{T} \end{array}$$
c. Determine the Hessian Matrix H(θ)
  $$H\left(\theta \right)=-\frac{1}{n}\left[q^{T}\left(\theta \right)q\left(\theta \right)\right]$$

The BHHH algorithm is formulated in Eq. (12).

(12) $${\hat{\theta }}^{\left(t+1\right)}={\hat{\theta }}^{\left(t\right)}-H^{-1}\left({\hat{\theta }}^{\left(t\right)}\right)q\left({\hat{\theta }}^{\left(t\right)}\right);\;t=0,1,2,...,T$$

If the convergence condition is $∥{\hat{\theta }}^{\left(t+1\right)}-{\hat{\theta }}^{\left(t\right)}∥\leq \varepsilon$, then the iteration will end at the *T*-th iteration where ε is error value. In this study, the optimal polynomial degree and the best model are obtained using Deviance criteria, which is defined as:

(13) $$Deviance=-2\ln L\left(\hat{\theta }\right)$$

where $L(\hat{\theta })$ is the maximum-likelihood. The optimal polynomial degree and the best model is obtained by the minimum values of Deviance.

### Statistical testing of the BPBLR model parameter

In the BPBLR model, simultaneous statistical testing is carried out by the Maximum Likelihood Ratio Test (MLRT) method [25]. The following is the hypothesis:

$$\begin{array}{ccc} & & H_{0}:\beta_{p1q}=\beta_{p2q}=\gamma_{pq}=0;p=1,2,...,r_{q};q=1,2,...,k\\ & & H_{1}:\text{at}\text{least}\text{one}\beta_{p1q}\neq 0\text{or}\beta_{p2q}\neq 0\text{or}\gamma_{pq}\neq 0 \end{array}$$

The first step to obtaining the maximized likelihood function under $H_{0}$, L(ω), is to define the parameters under $H_{0}$, $\omega =\{\alpha_{1},\alpha_{2},\alpha_{3}\}$. Therefore, the BPBLR model under $H_{0}$ is:

(14) $$\begin{array}{ccc} & & ln\left(\frac{\pi_{1}}{1-\pi_{1}}\right)=\alpha_{1}\\ & & ln\left(\frac{\pi_{2}}{1-\pi_{2}}\right)=\alpha_{2}\\ & & ln\left(\frac{\pi_{00}\pi_{11}}{\pi_{01}\pi_{10}}\right)=\alpha_{3} \end{array}$$

The likelihood function under $H_{0}$ is:

(15) $$L\left(\omega \right)=\prod_{i=1}^{n}\left[{\left(\pi_{11i}^{*}\right)}^{y_{11i}}{\left(\pi_{10i}^{*}\right)}^{y_{10i}}{\left(\pi_{01i}^{*}\right)}^{y_{01i}}{\left(\pi_{00i}^{*}\right)}^{y_{00i}}\right]$$

The natural logarithm likelihood function under $H_{0}$ is:

(16) $$\ln L\left(\omega \right)=\sum_{i=1}^{n}\left(y_{11i}\ln\pi_{11i}^{*}+y_{10i}\ln\pi_{10i}^{*}+y_{01i}\ln\pi_{01i}^{*}+y_{00i}\ln\pi_{00i}^{*}\right)$$

Based on the MLE method and the BHHH iteration, the parameter estimator was obtained, ${\hat{\alpha }}_{1},{\hat{\alpha }}_{2},{\hat{\alpha }}_{3}$. Furthermore, the estimated probability value, i.e. ${\hat{\pi }}_{11i}^{*},{\hat{\pi }}_{10i}^{*},{\hat{\pi }}_{01i}^{*},\text{and}\;{\hat{\pi }}_{00i}^{*}$, can be obtained as follows:

(17) $${\hat{\pi }}_{11i}^{*}=\frac{a_{1}-\sqrt{a_{1}^{2}+b_{1}}}{2\left({\hat{\psi }}_{1}-1\right)}$$

with,

$a_{1}=1+\left({\hat{\psi }}_{1}-1\right)\left({\hat{\pi }}_{1i}^{*}+{\hat{\pi }}_{2i}^{*}\right)$; $b_{1}=-4{\hat{\psi }}_{1}\left({\hat{\psi }}_{1}-1\right){\hat{\pi }}_{1i}^{*}{\hat{\pi }}_{2i}^{*}$; ${\hat{\psi }}_{1}=\exp\left({\hat{\alpha }}_{3}\right)$.

Based on Eq. (14), we can obtain ${\hat{\pi }}_{1i}^{*}$ and ${\hat{\pi }}_{2i}^{*}$. Furthermore, we can get ${\hat{\pi }}_{10i}^{*}$, ${\hat{\pi }}_{01i}^{*}$ and ${\hat{\pi }}_{00i}^{*}$ based on Eq. (5). Therefore, we can get the maximized natural logarithm likelihood function under $H_{0}$ is:

(18) $$\ln L\left(\hat{\omega }\right)=\sum_{i=1}^{n}\left(y_{11i}\ln{\hat{\pi }}_{11i}^{*}+y_{10i}\ln{\hat{\pi }}_{10i}^{*}+y_{01i}\ln{\hat{\pi }}_{01i}^{*}+y_{00i}\ln{\hat{\pi }}_{00i}^{*}\right)$$

Next, the set of parameters under population is $\Omega =\{\alpha_{1},\alpha_{2},\alpha_{3},\beta_{p1q},\beta_{p2q},\gamma_{pq};p=1,2,...,r_{q},$ q=1,2,...,k}.

Therefore, the likelihood function of Eq. (18) is formulated by:

(19) $$L\left(\Omega \right)=\prod_{i=1}^{n}\left\{{\left(\pi_{11i}\right)}^{y_{11i}}{\left(\pi_{10i}\right)}^{y_{10i}}{\left(\pi_{01i}\right)}^{y_{01i}}{\left(\pi_{00i}\right)}^{y_{00i}}\right\}$$

The natural logarithm likelihood function:

(20) $$\ln L\left(\Omega \right)=\sum_{i=1}^{n}\left(y_{11i}\ln\pi_{11i}+y_{10i}\ln\pi_{10i}+y_{01i}\ln\pi_{01i}+y_{00i}\ln\pi_{00i}\right)$$

The estimated probability value, i.e ${\hat{\pi }}_{11i},{\hat{\pi }}_{10i},{\hat{\pi }}_{01i},\text{and}\;{\hat{\pi }}_{00i}$, are formulated as follows:

(21) $${\hat{\pi }}_{11i}=\frac{a_{2}-\sqrt{a_{2}^{2}+b_{2}}}{2\left({\hat{\psi }}_{2}-1\right)}$$

With,

$a_{2}=1+\left({\hat{\psi }}_{2}-1\right)\left({\hat{\pi }}_{1i}+{\hat{\pi }}_{2i}\right)$; $b_{2}=-4{\hat{\psi }}_{2}\left({\hat{\psi }}_{2}-1\right){\hat{\pi }}_{1i}{\hat{\pi }}_{2i}$; ${\hat{\psi }}_{2}=\exp\left({\hat{\alpha }}_{3}+\sum_{j=1}^{k}{\hat{\gamma }}_{j}^{T}x_{j(r_{j})}^{*}\right)$

Based on Eq (20), we can obtain ${\hat{\pi }}_{1i}$ and ${\hat{\pi }}_{2i}$with ${\hat{\beta }}_{1i}$ and ${\hat{\beta }}_{2j}$ are maximum likelihood estimators for $\beta_{1i}$ and $\beta_{2j}$. Furthermore, we can get ${\hat{\pi }}_{10i}$, ${\hat{\pi }}_{01i}$ and ${\hat{\pi }}_{00i}$ using Eq. (5). Therefore, we get the maximized likelihood function under population:

(22) $$\ln L\left(\hat{\Omega }\right)=\sum_{i=1}^{n}\left(y_{11i}\ln{\hat{\pi }}_{11i}+y_{10i}\ln{\hat{\pi }}_{10i}+y_{01i}\ln{\hat{\pi }}_{01i}+y_{00i}\ln{\hat{\pi }}_{00i}\right)$$

The statistical testing based on MLRT method is formulated by

(23) $$G^{2}=-2\ln\left(\frac{L\left(\hat{\omega }\right)}{L\left(\hat{\Omega }\right)}\right)=2\left(\ln L\left(\hat{\Omega }\right)-\ln L\left(\hat{\omega }\right)\right)$$

where $\ln L(\hat{\omega })$ from Eq. (20) and $\ln L(\hat{\Omega })$ from Eq. (22). Next, Eq. (23) can be obtained as follows

(24) $$G^{2}=2\sum_{i=1}^{n}\left[y_{11i}\ln\left(\frac{{\hat{\pi }}_{11i}}{{\hat{\pi }}_{11i}^{*}}\right)+y_{10i}\ln\left(\frac{{\hat{\pi }}_{1i}-{\hat{\pi }}_{11i}}{{\hat{\pi }}_{1i}^{*}-{\hat{\pi }}_{11i}^{*}}\right)+y_{01i}\ln\left(\frac{{\hat{\pi }}_{2i}-{\hat{\pi }}_{11i}}{{\hat{\pi }}_{2i}^{*}-{\hat{\pi }}_{11i}^{*}}\right)+y_{00i}\ln\left(\frac{1-{\hat{\pi }}_{1i}-{\hat{\pi }}_{2i}+{\hat{\pi }}_{11i}}{1-{\hat{\pi }}_{1i}^{*}-{\hat{\pi }}_{2i}^{*}+{\hat{\pi }}_{11i}^{*}}\right)\right]$$

The test statistic $G^{2}$ follows the asymptotic of the Chi-square limit distribution and the degrees of freedom is $v=\left(3+3\sum_{j=1}^{k}r_{j}\right)-\left(3\right)=3\sum_{j=1}^{k}r_{j}$. Therefore, reject $H_{0}$ when $G^{2}>\chi_{\left(\alpha ,v\right)}^{2}$, where $\chi_{\left(\alpha ,v\right)}^{2}$ is the Chi-square distribution with α significant level and *v* degree of freedom.

## Method validation

The BPBLR model was applied to modeling of the depth and severity of poverty in districts/cities on the Java island in 2022. The data was obtained from the Badan Pusat Statistik (BPS) of 6 provinces on the Java island, i.e. DKI Jakarta, Banten, West Java, Central Java, DI Yogyakarta and East Java. The data taken from BPS for each province is published data which includes Regency/City Poverty Data and Information in Indonesia in 2022, Provinces in Numbers, and Regency/City Gross Regional Domestic Product Reports in Indonesia for 2018–2022. The research units are districts/cities from 6 provinces on the Java island in 2022 consisting of 85 districts and 34 cities so that the total number of research units is 119 districts/cities.

There are two binary response variables and five predictor variables in this study. The response variables are the depth of poverty (Y1) and the severity of poverty (Y2) in districts/cities on the Java-island in 2022. Each response variable has two categories (binary), i.e. low and high. If the district/city index value is less than the Indonesian index value then it is categorized as low and denoted by 0, whereas if the district/city index value is more than or equal to the Indonesian index value then it is categorized as high and denoted by 1. The predictor variables are factors which is thought to influence the depth and severity of poverty in each district/city on the Java-island in 2022 which includes the open unemployment rate (X1), the literacy rate of the population aged ≥ 15 years (X2), the average number of years of schooling (X3), the middle age school participation (X4) and GDP growth rate at constant prices (X5). The results of the description of the response variable are presented in Table 2.

**Table 2:** Description of the depth and severity of poverty.

	$Y_{2}=0$	$Y_{2}=1$	Total
$Y_{1}=0$	73	5	78
$Y_{1}=1$	7	34	41
Total	80	39	119

**Fig. 1:**
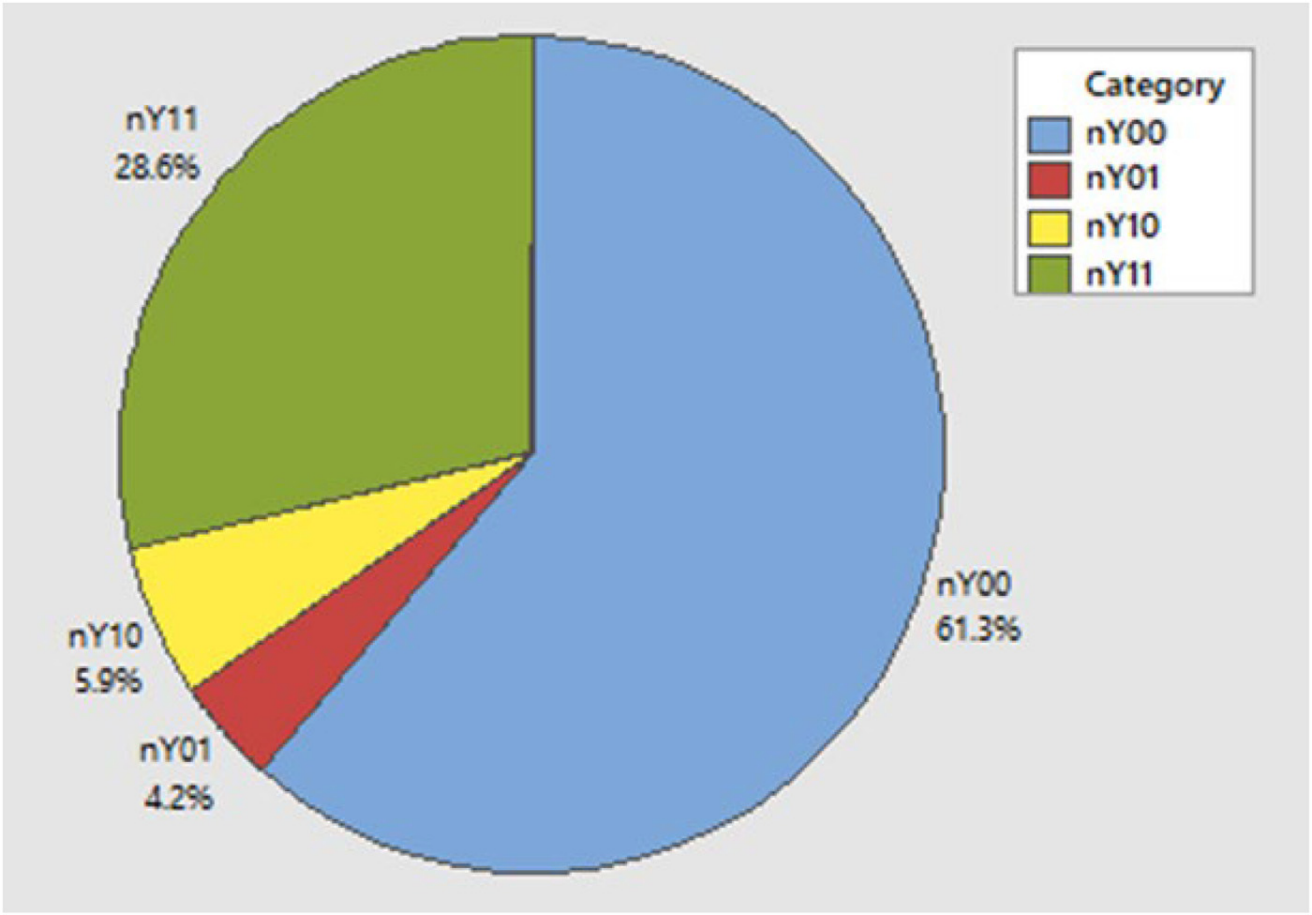
Description of the percentage level of depth and severity of poverty.

Table 2 shows that there are 34 districts/cities that have a high level of depth and severity of poverty on the Java Island in 2022 (Y_11_). Furthermore, there are 5 districts/cities that have a low level of poverty depth and a high level of poverty severity (Y_01_). There are 7 districts/cities that have a high level of depth of poverty and a low level of poverty severity (Y_10_). Meanwhile, 73 districts/cities have a low level of depth and severity of poverty (Y_00_). A description of the depth and severity of poverty in districts/cities on the Java-island in 2022 can also be presented in Fig. 1.

Based on Fig. 1, the number of districts/cities that have a high level of depth and severity of poverty (Y_11_) is 28.6 %. The number of districts/cities that have a low level of poverty depth and a high level of poverty severity (Y_01_) is 4.2 %. The number of districts/cities with high poverty depth and low poverty severity (Y_10_) is 5.9 % and the number of districts/cities with low poverty depth and severity (Y_00_) is 61.3 %. Next, description of the predictor variables is given in Table 3*.*

**Table 3:** Description of predictor variables.

Variable	Minimum	Maximum	Mean	Standard Deviation
X_1_	1.36	10.78	6.124	2.279
X_2_	86.03	100	98.001	2.342
X_3_	5.06	11.89	8.6	1.604
X_4_	54.42	100	93.886	8.811
X_5_	-6.7	8.3	4.757	1.738

It is shown in Table 3 that the average open unemployment rate for districts/cities on the Java island in 2022 is 6.124 % with the highest percentage in Bogor City, West Java at 10.78 % while the lowest percentage is in Sumenep Regency, East Java at 1.36 %. The literacy rate for residents aged ≥ 15 years on the Java island in 2022 will average 98,001 % and there are 33 districts/cities that have a literacy rate for residents aged ≥ 15 years of 100 %, while the district/city that has the lowest percentage is Situbondo Regency, East Java amounted to 86.03 %. The average length of schooling on the Java island in 2022 is 8.6 years. Sampang Regency, East Java has the lowest average length of school, i.e. 5.06 years, while Yogyakarta City has the highest average length of school, i.e. 11.89 years. The average middle school enrollment rate on the Java island in 2022 is 93.886 % and there are 58 districts/cities that have a middle age school participation rate of 100 %, while the district/city that has the lowest percentage is Serang City, Banten at 54.42 %. The average GRDP growth rate at constant prices in districts/cities on Java Island in 2022 is 4757 % with the highest percentage in Tuban Regency, East Java and the lowest percentage in Bojonegoro Regency, East Java.

Furthermore, the multicollinearity test was carried out with the aim of finding out whether a correlation was found in a regression model between the predictor variables. In this study, the multicollinearity test using the Variance Inflation Factor (VIF) value criterion. Table 4 provides the value of the VIF for each of the predictor variables as follows.

**Table 4:** VIF for each predictor variable.

Variable	VIF Value
X_1_	1.303101
X_2_	1.398406
X_3_	1.479728
X_4_	1.130071
X_5_	1.099989

Table 4 indicates that all predictor variables have VIF values less than 10. Therefore, it can be interpreted that multicollinearity does not occur. Thus, all predictor variables can be used for further analysis.

The next step is testing independence between the response variables, which aims to determine whether the two response variables depend on each other so that they are suitable for bivariate analysis. The Chi-Square test was used for the testing and Table 5 provides the results.

**Table 5:** Result of response variable dependency test.

Statistical Tests	Chi-Square	df	p-value
Pearson	71.414	1	0.000
Likelihood Ratio	75.927	1	0.000

Table 5 indicates that there is a dependency between the depth of poverty and the severity of poverty on the Java island in 2022. The depth and the severity of poverty used as response variables in this study are suitable for use in the BPBLR model analysis.

Modeling the depth and severity of poverty in districts/cities on the Java island in 2022 based on the BPBLR model begins with selecting predictor variables that have an individual significant effect on the response variable by regressing each predictor variable on the response variable. This aims to find out which predictor variables univariably have a significant effect on the response variable. The results of the univariable BPBLR model analysis are presented in Table 6 below:

**Table 6:** Results of univariable BPBLR model analysis.

Predictor Variable	Degree of Polynomials	G^2^	df	*P-Value*
X_1_	1	5.488875	3	0.1393055
	2	8.729121	6	0.189395
X_2_	1	5.402372	3	0.144596
	2	844.3672	6	0.0000000⁎
X_3_	1	28.25181	3	0.0000032⁎
	2	30.80155	6	0.0000277⁎
X_4_	1	2.366246	3	0.4999488
	2	844.1036	6	0.0000000⁎
X_5_	1	8.060675	3	0.0447743⁎
	2	12.82368	6	0.0459230⁎

⁎ Significant at α=5%.

Table 6 shows that there are four predictor variables, at either polynomial degree 1 or 2, univariably have a significant impact on the response variables, i.e X2, X3, X4 and X5.

The next step is to construct a Multivariable BPBLR model. This model involves four significant predictor variables from the results of univariable analysis. Modeling is carried out by regressing the four predictor variables together on the response variable for several combinations of polynomial degrees. Next, the best model is selected based on the optimal polynomial degree of each predictor variable which produces the minimum Deviance. The Deviance values of the multivariable BPBLR model for all combinations of polynomial degrees are presented in Table 7.

**Table 7:** Deviance values of the BPBLR model.

No	Degrees of X_2_ ($r_{2}$)	Degrees of X_3_ ($r_{3}$)	Degrees of X_4_ ($r_{4}$)	Degrees of X_5_($r_{5}$)	*Deviance*
1.	1	1	1	1	186.656
2.	2	1	1	1	183.221
3.	1	2	1	1	202.944
4.	2	2	1	1	209.416
5.	1	1	2	1	191.242
6.	2	1	2	1	198.935
7.	1	2	2	1	217.309
8.	2	2	2	1	215.716
9.	1	1	1	2	190.617
10.	2	1	1	2	181.177
11.	1	2	1	2	193.200
12.	2	2	1	2	197.975
13.	1	1	2	2	187.169
14.	2	1	2	2	196.900
15.	1	2	2	2	212.706
16.	2	2	2	2	211.755

Based on Table 7, the minimum Deviance value is 181.177. So, the best BPBLR model which has the minimum Deviance value is obtained when r_2_ = 2, r_3_ = 1, r_4_ = 1 and r_5_ = 2. Next, the BPBLR model parameter estimation was carried out for the best model using BHHH numerical iteration and the results are presented in Table 8 below:

**Table 8:** The results of BPBLR model parameter estimation for the best model.

No.	Parameter	Estimation	SE	*P-Value*
1.	$\alpha_{1}$	-734.03	0.0000701	0.000000⁎
2.	$\beta_{112}$	15.44	0.0033590	0.000000⁎
3.	$\beta_{212}$	-0.08	0.0002338	0.000000⁎
4.	$\beta_{113}$	-0.93	0.0008446	0.000000⁎
5.	$\beta_{114}$	-0.00	0.0204962	0.879092
6.	$\beta_{115}$	0.13	0.0010958	0.000000⁎
7.	$\beta_{215}$	0.00	0.0161163	0.853205
8.	$\alpha_{2}$	-451.74	0.0000575	0.000000⁎
9.	$\beta_{122}$	9.45	0.0027688	0.000000⁎
10.	$\beta_{222}$	-0.05	0.0002450	0.000000⁎
11.	$\beta_{123}$	-0.85	0.0008451	0.000000⁎
12.	$\beta_{124}$	-0.01	0.0226140	0.740743
13.	$\beta_{125}$	0.56	0.0017421	0.000000⁎
14.	$\beta_{225}$	-0.04	0.0172125	0.012545⁎
15.	$\alpha_{3}$	1596.77	0.0000072	0.000000⁎
16.	$\gamma_{12}$	-36.61	0.0003405	0.000000⁎
17.	$\gamma_{22}$	0.20	0.0002003	0.000000⁎
18.	$\gamma_{13}$	-8.34	0.0000766	0.000000⁎
19.	$\gamma_{14}$	1.06	0.0012269	0.000000⁎
20.	$\gamma_{15}$	17.78	0.0002394	0.000000⁎
21.	$\gamma_{25}$	-1.40	0.0019629	0.000000⁎
Likelihood Ratio Test ($G^{2}$) = 454.1906, *df* = 18, p-value = 0.000000⁎				

⁎ Significant at α=5%.

In the BPBLR model, parameter testing is carried out simultaneously and partially. The hypotheses used for simultaneous testing is given in Eq (4.1). The value of $G^{2}$ statistical test in Table 8 is 454.1906. Whereas a value of Chi-square table with 5 % significant level and 18 degrees of freedom is 28.8693. The value of $G^{2}$ is greater than the value of Chi-square table, then reject H_0_. So, it can be concluded that there is minimum one predictor variable that significantly influences on the depth of poverty (Y_1_) and the severity of poverty (Y_2_) on the Java island in 2022.

Next, partial parameter testing is used to identify which predictor variables that are significant to the model. Table 8 indicates the predictor variables that significantly affect the depth of poverty (Y_1_) and the severity of poverty (Y_2_) based on the BPBLR model are the literacy rate of the population aged ≥ 15 years (X_2_), the average number of years of schooling (X_3_) and GDP growth rate at constant prices (X_5_). Furthermore, based on the results in Table 8, the BPBLR model formed is as follows:

Logit model 1:

$\ln\left(\frac{\pi_{1}}{1-\pi_{1}}\right)=-734.03+15.44X_{2}-0.08X_{2}^{2}-0.93X_{3}+0.13X_{5}$

Logit model 2:

$\ln\left(\frac{\pi_{2}}{1-\pi_{2}}\right)=-451.74+9.45X_{2}-0.05X_{2}^{2}-0.85X_{3}+0.56X_{5}-0.04X_{5}^{2}$

Odds ratio transformation model:

$\ln\left(\frac{\pi_{00}\pi_{11}}{\pi_{01}\pi_{10}}\right)=1596.77-36.61X_{2}+0.2X_{2}^{2}-8.34X_{3}+1.06X_{4}+17.78X_{5}-1.4X_{5}^{2}$

Y_1_ marginal opportunity model:

$\pi_{1}=\frac{\exp(-734.03+15.44X_{2}-0.08X_{2}^{2}-0.93X_{3}+0.13X_{5})}{1+\exp(-734.03+15.44X_{2}-0.08X_{2}^{2}-0.93X_{3}+0.13X_{5})}$

Y_2_ marginal opportunity model:

$\pi_{2}=\frac{\exp(-451.74+9.45X_{2}-0.05X_{2}^{2}-0.85X_{3}+0.56X_{5}-0.04X_{5}^{2})}{1+\exp(-451.74+9.45X_{2}-0.05X_{2}^{2}-0.85X_{3}+0.56X_{5}-0.04X_{5}^{2})}$

Based on the logit model 1, it can be interpreted that if the percentage of literacy rate of the population aged ≥ 15 years increases by one unit, the ratio (odds) for high poverty levels will increase by exp(15.44) times compared to low poverty levels. Meanwhile, if the average percentage of years of schooling increases by one unit, the ratio (odds) for high levels of poverty depth will decrease by exp(0.93) times that of low levels of poverty depth and if the GDP growth rate at constant prices increases by one unit, the ratio (odds) a high level of poverty depth will increase by exp(0.13) times than a low level of poverty depth.

Based on the logit model 2, it can be interpreted that if the percentage of literacy rate of the population aged ≥ 15 years increases by one unit, the ratio (odds) for the severity of high poverty will increase by exp(9.45) times compared to the severity of low poverty. Meanwhile, if the average percentage of years of schooling increases by one unit, the ratio (odds) for high levels of poverty severity will decrease by exp(0.85) times that for low levels of poverty severity and if the GDP growth rate at constant prices increases by one unit, the ratio (odds) High poverty severity levels will increase by exp(0.56) times than low poverty severity levels.

## Limitations

The BPBLR model is a development of binary logit regression for two correlated binary response and the association between the logit and predictor variables is described as a polynomial. The MLE method was applied to estimate the BPBLR model's parameters. The maximum likelihood estimators of the BPBLR model are not in explicit form and re-quires a numerical iterative method. The iterative BHHH was utilized. The MLRT method is used for the simultaneous statistical testing and asymptotically the distribution of the statistical test is Chi-squared. The optimal polynomial degree of predictor variable is obtained based on the minimum Deviance value.

## Declaration of competing interest

The authors declare that they have no known competing financial interests or personal relationships that could have appeared to influence the work reported in this paper.

## Data Availability

Data will be made available on request.
